# Painting authentication using CNNs and sliding window feature extraction

**DOI:** 10.3389/frai.2025.1738444

**Published:** 2026-01-13

**Authors:** Juan Ruiz de Miras, José Luis Vílchez, María José Gacto, Domingo Martín

**Affiliations:** 1 Software Engineering Department, University of Granada, Granada, Spain; 2 Department of Analytical Chemistry, University of Granada, Granada, Spain; 3 Biosanitary Research Institute (ibs.GRANADA), Granada, Spain; 4 Andalusian Research Institute in Data Science and Computational Intelligence (DaSCI), University of Granada, Granada, Spain

**Keywords:** authentication, convolutional neural networks, painting classification, Paolo Veronese, sliding-window patch extraction

## Abstract

Painting authentication is an inherently complex task, often relying on a combination of connoisseurship and technical analysis. This study focuses on the authentication of a single painting attributed to Paolo Veronese, using a convolutional neural network approach tailored to severe data scarcity. To ensure that stylistic comparisons were based on artistic execution rather than iconographic differences, the dataset was restricted to paintings depicting the Holy Family, the same subject as the work under authentication. A custom shallow convolutional network was developed to process multichannel inputs (RGB, grayscale, and edge maps) extracted from overlapping patches via a sliding-window strategy. This patch-based design expanded the dataset from a small number of paintings to thousands of localized patches, enabling the model to learn microtextural and brushstroke features. Regularization techniques were employed to enhance generalization, while a painting-level cross-validation strategy was used to prevent data leakage. The model achieved high classification performance (accuracy of 94.51%, Area under the Curve 0.99) and generated probability heatmaps that revealed stylistic coherence in authentic Veronese works and fragmentation in non-Veronese paintings. The work under examination yielded an intermediate global mean Veronese probability (61%) with extensive high-probability regions over stylistically salient passages, suggesting partial stylistic affinity. The results support the use of patch-based models for stylistic analysis in art authentication, especially under domain-specific data constraints. While the network provides strong probabilistic evidence of stylistic affinity, definitive attribution requires further integration with historical, technical, and provenance-based analyses.

## 1 Introduction

Art authentication remains a complex, multidisciplinary challenge that requires the integration of historical expertise, scientific analysis, and, increasingly, computational techniques (King, 2024). In recent years, deep learning has emerged as a powerful tool for image-based analysis, offering scalable, objective approaches that complement traditional expert judgment. Convolutional neural networks (CNNs) and other advanced architectures have demonstrated high levels of accuracy in art authentication by learning complex visual patterns directly from images.

However, despite the diversity of architectures and methodologies employed in prior work (see Section 2), deep-learning approaches share a fundamental requirement: access to large, labeled datasets for model training. Previous studies rely on hundreds or thousands of high-resolution images of authenticated artworks to enable neural networks to learn meaningful stylistic and compositional patterns. This dependence on extensive training data remains a key limitation, particularly when authenticating a single artwork, where the training set of verified examples may consist of only a handful of paintings. This data-scarce scenario renders standard deep, pretrained models, which contain millions of parameters, highly prone to overfitting (Bejani and Ghatee, 2021). Moreover, transfer learning approaches often rely on features learned from natural images (e.g., ImageNet), which emphasize high-level semantic content over low-level microtextural nuances required to distinguish a master from a workshop. A significant gap therefore exists for tailored methodologies capable of operating effectively under these severe data constraints, with emphasis on execution technique rather than semantic composition.

**Figure 1:**
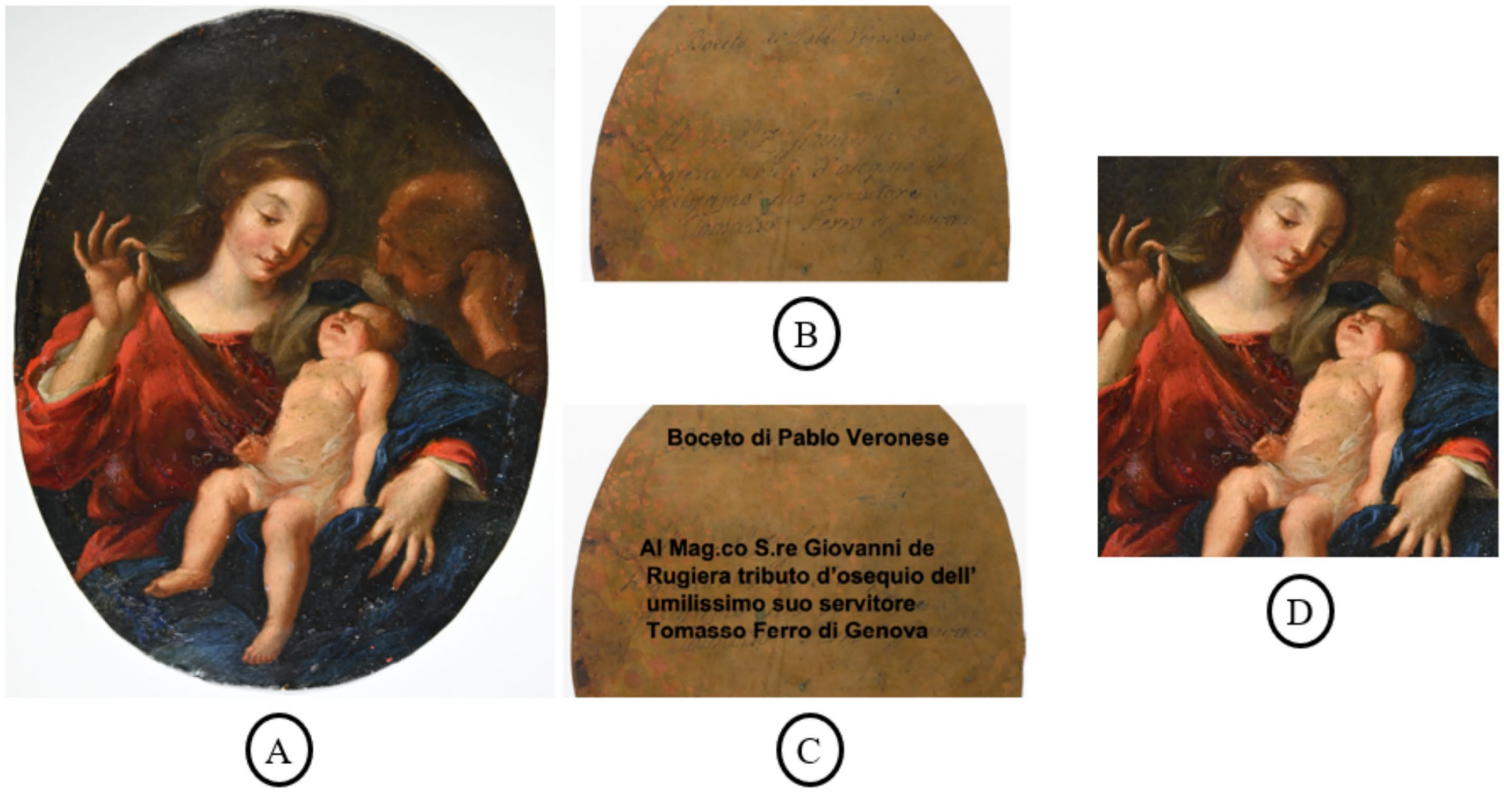
Holy Family painting under examination: **(A)** Original artwork, reproduced by the authors from “Holy Family: The Virgin”; **(B)** reverse with inscription; **(C)** reverse with highlighted inscription; and **(D)** cropped, squared image prepared for CNN analysis.

To address this gap, an approach based on a custom, shallow CNN and sliding-window-based feature extraction is introduced and applied to the authentication of a single painting with a disputed attribution. This patch-based approach mitigates data scarcity by substantially expanding the training set, shifting the model’s focus from global composition to learning localized, microstylistic features such as brushwork and texture (Sabha et al., 2024). Furthermore, the proposed model integrates multichannel inputs (RGB, grayscale, and edge maps) providing complementary visual cues; this technique has been shown to enhance the capture of subtle stylistic features (Ugail et al., 2023). The artwork, a Holy Family (see Figure 1A), has been linked to Paolo Veronese, although its authorship remains uncertain, and may originate from the master, Veronese’s workshop, or his disciples (Blanc et al., 2023; López-Baldomero et al., 2023). Training on a restricted dataset of Holy Family paintings attributed to Veronese and his circle, is intended to identify stylistic features that may clarify the painting’s origin, offering a reproducible and data-driven complement to expert judgment.

The main contributions of this study are as follows:

- A tailored deep-learning framework for severe data scarcity: a sliding-window patch-generation strategy combined with a custom, shallow CNN architecture that effectively mitigates overfitting, enabling robust training with a dataset of only six paintings.
- Multichannel stylistic feature extraction: integrating RGB, grayscale, and edge maps enables the model to capture complementary stylistic cues, such as brushwork texture and structural contours, that are critical for distinguishing a master from a workshop or circle.
- Painting-level validation: the implementation of a painting-level leave-one-out cross-validation strategy (rather than at the patch level) ensures unbiased performance estimation and prevents data leakage.
- Quantitative evidence for a disputed attribution: a probabilistic assessment of the Holy Family painting under examination is provided, offering objective data that support the hypothesis of partial stylistic affinity.

The remainder of this paper is organized as follows: Section 2 reviews related work; Section 3 describes the dataset and the proposed methodology; Section 4 presents the experimental results; and Section 5 discusses the results and presents the conclusions.

## 2 Related work

Deep learning has emerged as a powerful approach for image-based analysis in the cultural-heritage domain. CNNs and advanced architectures, such as vision transformers, have demonstrated high levels of accuracy in art authentication by learning complex visual patterns directly from images (Elgammal et al., 2018; Dobbs et al., 2023; Ugail et al., 2023; Chen et al., 2024; Schaerf et al., 2024).

Specifically, in Schaerf et al. (2024), the use of vision transformers for the authentication of Van Gogh paintings was explored, showing that deep-learning models can outperform handcrafted-feature approaches in both precision and interpretability. Similarly, Dobbs applied large-scale CNN-based classification framework to contemporary artworks, achieving more than 91% accuracy and highlighting deep learning’s potential for scalable authentication across diverse artistic styles (Dobbs et al., 2023).

In Ugail et al. (2023), the effectiveness of transfer learning in attributing Renaissance paintings, specifically works by Raphael, using pretrained CNNs fine-tuned on curated datasets was demonstrated. Their approach provided valuable insights into stylistic attribution and supported expert judgment in complex cases. The study in Chen et al. (2024) further reinforced the utility of CNNs in fine-art recognition, emphasizing their role in extracting stylistic and compositional features for authentication tasks. Earlier work in Elgammal et al. (2018) introduced a deep-learning framework for analyzing stylistic evolution in art history, laying the groundwork for subsequent applications in authentication and attribution.

Beyond the cultural-heritage domain, the efficacy of shallow and custom-designed CNNs has been demonstrated in other fields requiring high levels of precision under specific constraints. Recent advances show that compact models, when properly optimized, can rival deeper architectures. For instance, Radojcic proposed a two-layer TinyML approach for plant disease classification (Radojcic et al., 2026), highlighting the computational efficiency of shallow networks. Similarly, the studies in Zivkovic et al. (2025) and Basha et al. (2021) employed metaheuristic optimization strategies to enhance CNN design for ocular-disease diagnosis and general-purpose applications, respectively. These studies reinforce the premise that tailored, shallow architectures constitute a robust solution for specialized tasks where massive datasets for pretraining are unavailable or unsuitable.

## 3 Materials and methods

### 3.1 Materials

The original painting under examination is shown in Figure 1A, while the cropped image used for analysis appears in Figure 1D. The crop was performed primarily to fit the elliptical composition into a rectangular frame, without introducing artificial background pixels that could bias the analysis. The resulting image was processed into square patches, a common input format for CNN analysis.

The painting depicts the Holy Family: The Virgin holding the Baby Jesus while lifting a veil, observed from behind by a figure identified as St. Joseph. An inscription is preserved on the reverse. The original text appears in Figure 1B, and the highlighted version in Figure 1C. This inscription may indicate authorship by P. Veronese, a copy after Paolo Veronese, or production by his workshop or followers. The painting is currently located in Spain and is part of a private collection.

The painting was previously analyzed using several analytical techniques (Blanc et al., 2023; López-Baldomero et al., 2023): X-ray fluorescence spectroscopy to identify chemical elements in the paint, X-ray diffraction to determine crystalline components of the pigments, and spectral image analysis with endmember extraction to identify pigments. These studies concluded that the materials and artistic techniques are consistent with those of Italian Renaissance artists. However, the painting’s precise authorship was not established.

**Figure 2:**
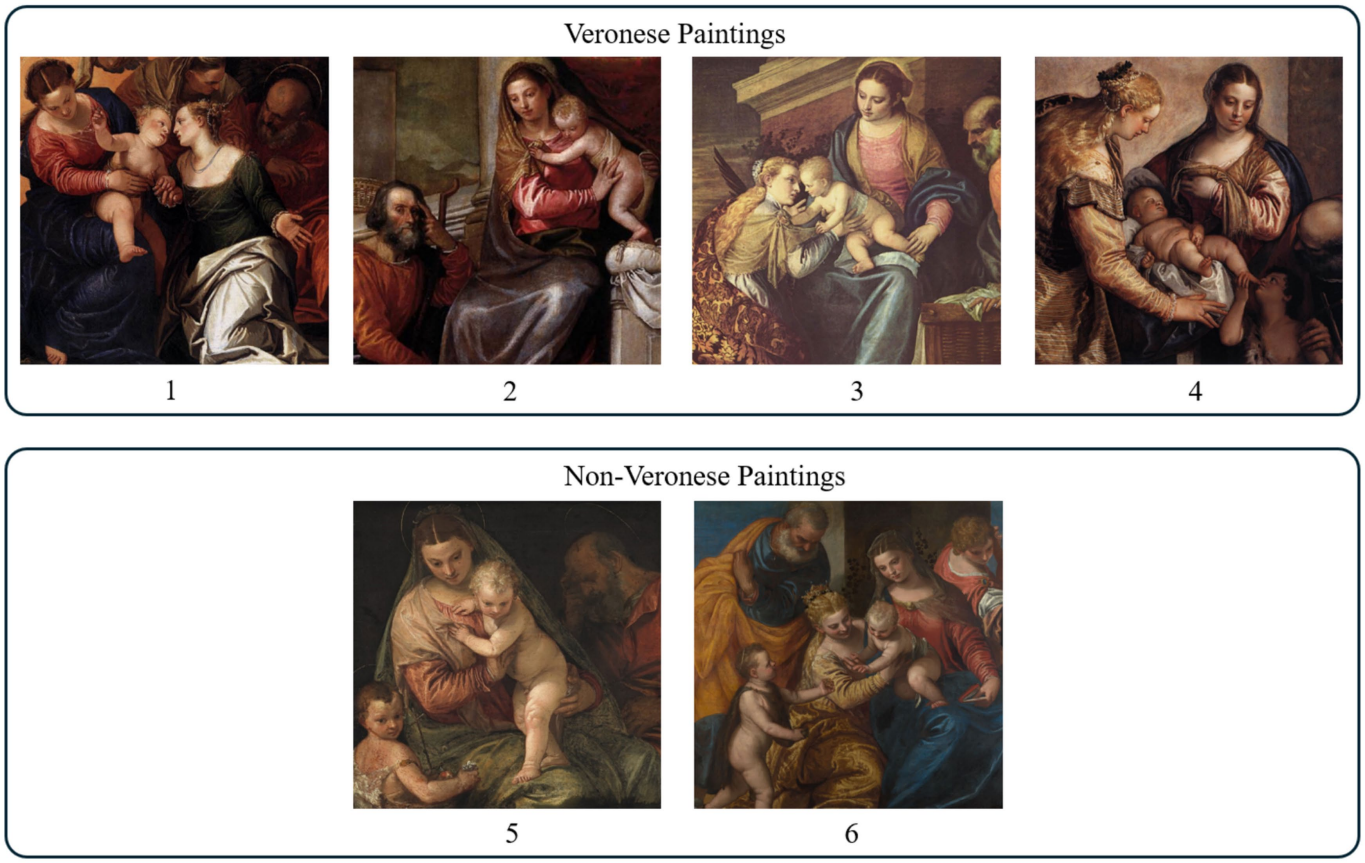
Training dataset of Holy Family paintings: (1–4) attributed to Paolo Veronese; (5, 6) attributed to his workshop or followers.

A set of Holy Family paintings (see Figure 2) was used to train the CNN. The training dataset was restricted to this subject to ensure thematic consistency with the work under examination. Maintaining thematic consistency, the CNN can focus on analyzing color palettes, visual patterns, and artistic techniques within a controlled, homogeneous context, thereby reducing variability unrelated to authorship and improving the reliability of stylistic analysis. The training dataset was also limited to works attributed to Paolo Veronese, his workshop, or followers. This restriction ensures stylistic coherence within the dataset, as including works by other Renaissance painters could introduce significant variability in composition, color schemes, and brushwork. Focusing exclusively on Veronese’s circle, the CNN can learn discriminative features specific to this artistic environment, thus reducing noise from unrelated stylistic traits and improving model’s ability to capture subtle patterns relevant to authorship attribution. Details for each painting are listed in Table 1. Four paintings (1–4) are confirmed works by Paolo Veronese (the Veronese class), and two paintings (5–6) are attributed to his disciples or followers (the non-Veronese class).

**Table 1:** Details for the paintings in the training dataset (1–6) and the test dataset (7–10).

Painting	Title	Author	Date	Additional Information
1	The Mystic Marriage of Saint Catherine of Alexandria	P. Veronese	1,547–50	https://artgallery.yale.edu/collections/objects/63825
2	Holy Family with Sts. Anthony Abbot, Catherine and the Infant John the Baptist	P. Veronese	1,551	https://www.wikiart.org/en/paolo-veronese/holy-family-with-sts-anthony-abbot-catherine-and-the-infant-john-the-baptist-1551
3	The Mystical Marriage of Saint Catherine	P. Veronese	1,557–65	https://www.museefabre.fr/recherche/musee%3AMUS_BIEN%3A3408?is_search_page=1&search=veronese&currentPage=1
4	Holy Family with Young St. John and St. Catherine	P. Veronese	1,565	https://www.uffizi.it/en/artworks/veronese-holy-family
5	The Holy Family with the Infant St. John the Baptist	Workshop of P. Veronese	1,550–75	https://id.rijksmuseum.nl/20026863
6	The Mystic Marriage of St. Catherine of Alexandria	Benedetto Caliari	1,562–9	https://www.rct.uk/collection/407216/the-mystic-marriage-of-st-catherine-of-alexandria
7	The Holy Family	Caravaggisti School	17th century	https://www.mutualart.com/Artwork/The-Holy-Family/AA108F3F26B11479DB06718BC7A2EB24
8	The Holy Family	Italian anonymous	17th century	http://fpjuliovisconti.com/anonimo-italiano-sagrada-familia-s-xvii/
9	–	Art student	21th century	–
10	Holy Family with Barbara	Reprodart.com	21th century	https://www.reprodart.com/a/veronese-paolo-eigentl-pa/pveroneseholyfamilywithba.html

A major challenge in this study is the limited size of the training dataset, which comprises only six paintings. Such a small sample poses a significant risk of overfitting and constrains a CNN’s ability to generalize effectively. To mitigate this constraint, a sliding-window method was implemented and applied to three distinct input representations (RGB, grayscale, and edge maps) for each painting, as detailed below. This approach substantially increased the number of training samples by generating many overlapping patches from each image, enhancing data diversity and enabling the model to learn local stylistic and textural features while preserving the overall artistic context.

**Figure 3:**
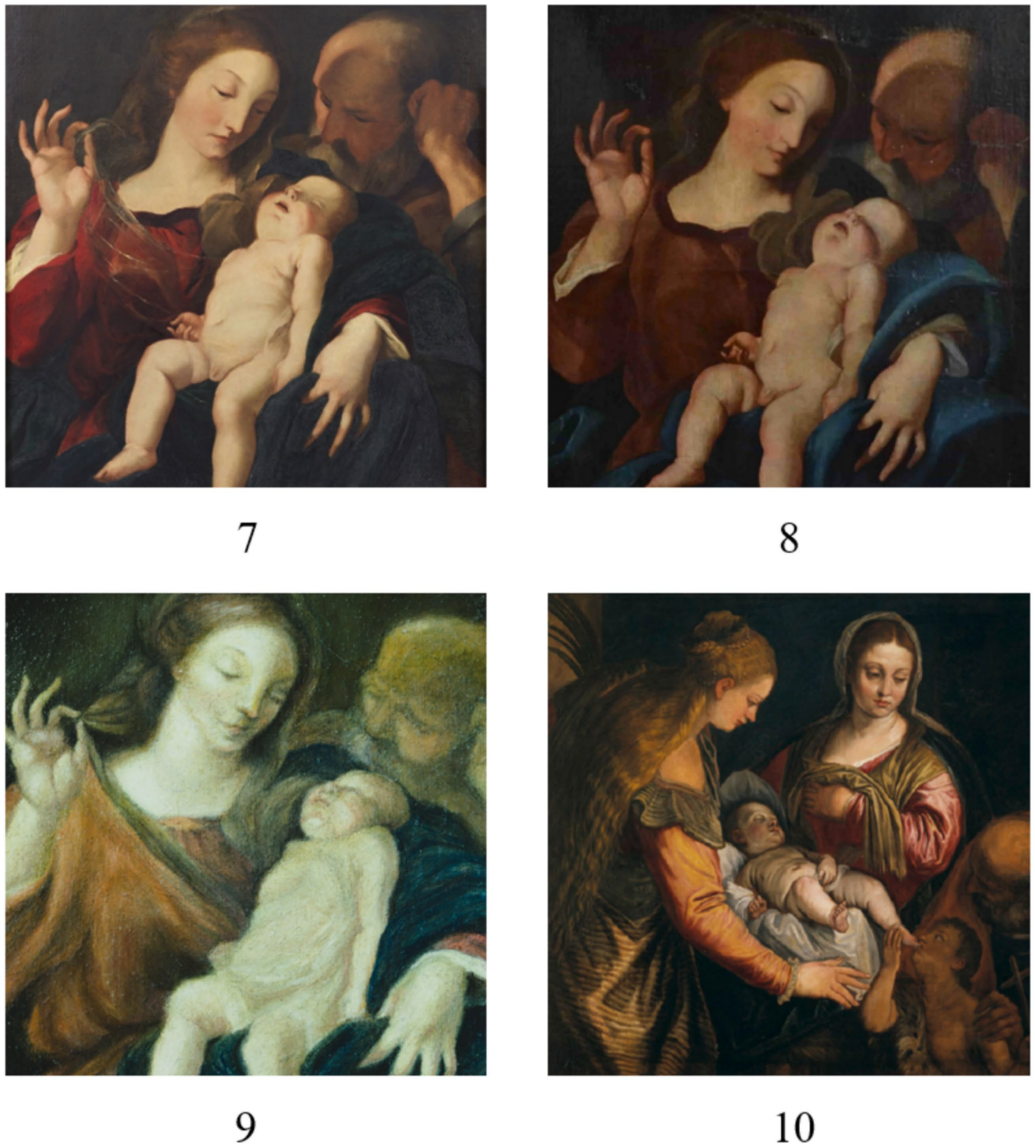
Test dataset of Holy Family paintings (paintings 7–10). Image 7 reproduced from “The Holy Family”, https://www.mutualart.com/Artwork/The-Holy-Family/AA108F3F26B11479DB06718; Image 8 reproduced from the Fundación Pintor Julio Visconti, “Anónimo Italiano Sagrada Familia, s. XVII”, http://fpjuliovisconti.com/anonimo-italiano-sagrada-familia-s-xvii/; Image 9 adapted by the authors from “The Holy Family”, https://www.mutualart.com/Artwork/The-Holy-Family/AA108F3F26B11479DB06718BC7A2EB24l; Image 10 reproduced from “Holy Family with St Barbara and the Infant St John” [c.1570] by Paolo Veronese, Holy Family with St Barbara and the Infant St John, c.1570 - Paolo Veronese - WikiArt.org, licensed under CC0.

CNN performance was evaluated using two distinct test sets. First, a negative test set was defined (see Figure 3; Table 1), comprising four Holy Family paintings with known non-Veronese authorship (Paintings 7, 8, 9, and 10). Paintings 7, 8, and 9 are copies of the work under examination: painting 7 is a 17th-century work by a Caravaggisti school artist; painting 8 is a work by an anonymous 17^th^-century Italian artist; and painting 9 is a modern copy created by an art student as a controlled experiment for this study. Painting 10 is a modern replica of Veronese’s painting 4. Second, to evaluate classification performance on authentic Veronese works, a painting-level leave-one-out cross-validation on Veronese paintings (Paintings 1–4) was conducted. Each Veronese painting (e.g., Painting 1) was held out as the test case, while training was performed on the remaining paintings (e.g., Paintings 2, 3, 4, 5, 6). This process was repeated, using each of paintings 1–4 as the test case in turn.

Images of all paintings by Veronese and his workshop, which are in the public domain, were downloaded from WikiArt - Visual Art Encyclopedia.^1^ The image for test painting 9 originated from the authors’ laboratory, while the images of paintings 7, 8, and 10 were obtained from the sources listed in Table 1. All images were cropped to square regions to include the entire Holy Family in each painting. These crops were downsampled to 640 × 640 pixels. This resolution was selected as the smallest square size capable of accommodating all figures, thus avoiding supersampling. The images used in the dataset can be accessed at https://www.ugr.es/~demiras/PaintingAuthentication/.

### 3.2 Methods

This section details the methodological pipeline: sliding-window based feature extraction (Section 3.2.1), the proposed CNN architecture (Section 3.2.2), and model validation and testing (Section 3.2.3).

#### 3.2.1 Sliding-window-based feature extraction from images of paintings

CNN training typically requires large, diverse datasets to ensure robust generalization and to mitigate overfitting. However, in the highly specialized context of painting authentication, the availability of labeled data is often severely limited, especially for rare or historically significant works. The training dataset comprises only six paintings, which poses a substantial challenge for conventional deep-learning models. CNNs trained on such small datasets tend to memorize the training samples rather than learn generalizable features, resulting in poor performance on unseen test data. This limitation has been acknowledged in the literature with studies exploring various strategies to mitigate overfitting, such as data augmentation, transfer learning, and feature extraction techniques (Sabha et al., 2024; Safa aldin et al., 2024). To address this issue, a sliding-window-based feature-extraction method was implemented. This technique produces a substantially larger set of localized patches, increasing the number of training samples and enhancing the CNN’s capacity to learn discriminative features.

**Figure 4:**
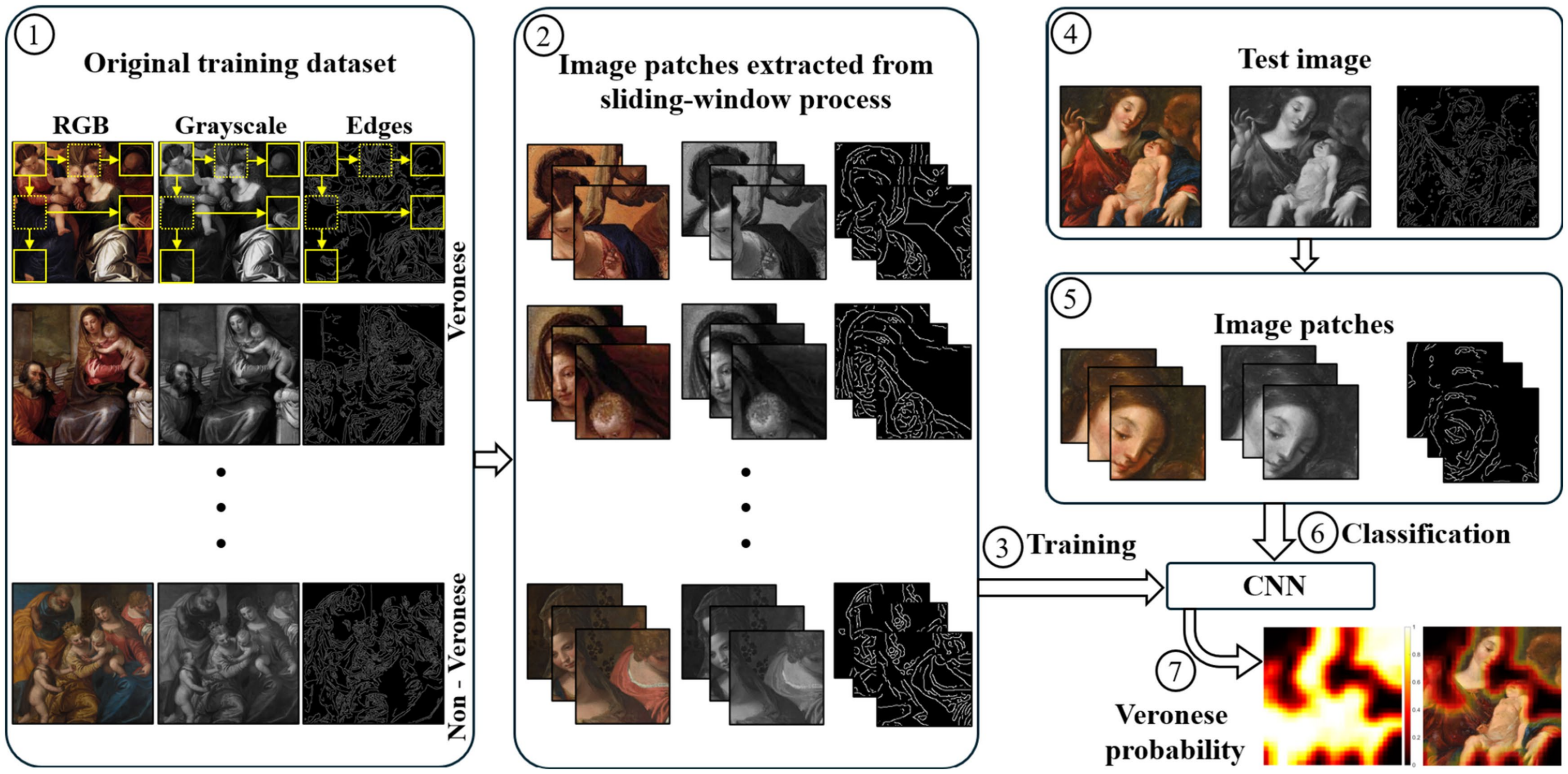
Proposed authentication methodology: (1) Original training dataset with application of the sliding-window technique; (2) Extraction of image patches via the sliding-window process to create an expanded training dataset; (3) CNN training on the expanded dataset; (4) Test image under authentication; (5) Extraction of image patches from the test image via the same sliding-window process; (6) CNN-based classification of all test patches; and (7) Generation of localized Veronese-probability maps (heatmap and overlay) and aggregation of patch-level classifications to estimate the painting’s global Veronese probability.

The sliding-window technique processes the input image by dividing it into fixed-size patches (windows) (see Figure 4, Step 1). The patches are obtained by moving the window across the image with a predefined step size, known as the stride. The stride can equal the patch dimensions or be smaller. When the stride is less than the patch size, the resulting patches exhibit a specified overlap, which is crucial for capturing continuous, fine-grained details and textures and generating a greater number of samples. Each patch inherits the source painting’s class label (“Veronese”/“Non-Veronese”; see Figure 4, Step 2).

This process is applied independently to three distinct input representations for each painting to maximize feature diversity (see Figure 4, Steps 1 and 2): (1) the original RGB image, (2) its grayscale version, and (3) a binary edge map. Use of the grayscale version enables the model to focus on structural and textural features without the influence of color, which can be particularly beneficial when color is not a reliable discriminative factor or under varying lighting conditions (Johnson et al., 2008). The edge map is generated with the Canny edge-detection algorithm (Canny, 1986). The Canny algorithm is particularly suitable because it detects edges by identifying local maxima of the image gradient using a dual-threshold mechanism. This dual-threshold approach classifies edges as strong or weak, including weak edges only if connected to a strong edge. This property makes the Canny algorithm more robust to noise than many alternatives and better at detecting subtle edges, which are critical for characterizing artistic style. This final, large collection of localized patches constitutes the expanded training dataset for the CNN (see Figure 4, Step 3).

The sliding window process has two parameters: (1) the window size, which determines the dimensions of the square patches extracted from the image, and (2) the overlap percentage, which determines the stride, i.e., the pixel shift between consecutive windows. These parameters directly influence the granularity of feature extraction and the total number of patches produced for training and testing. In this study, the sliding window size was fixed at 64 × 64 pixels, corresponding to one-tenth of each original image dimension (640 × 640 pixels). This configuration strikes an effective balance between capturing local details, such as brushstroke patterns and texture, and maintaining sufficient contextual information within each patch. Smaller windows can lead to excessive fragmentation and loss of structural cues, while larger windows may dilute fine-grained features critical for authentication. The overlap percentage (65%) was chosen experimentally by testing multiple values between 0 and 95% and selecting the value that yielded the best validation performance for the CNN. This overlap not only increases the number of training patches but also reduces boundary inconsistencies, as reported in prior work on image tiling for CNN-based analysis (An et al., 2020; Cira et al., 2024). The process was implemented by stacking the three representations (color, grayscale, and edge maps) to create a single 5-channel input: 3 channels for RGB, 1 channel for grayscale, and 1 channel for the edge map. This yielded 729 patches per painting. Consequently, the training dataset expanded from six original paintings to a total of 4,374 patches (729 patches per painting × 6 paintings), each with dimensions of 64 × 64 × 5 pixels.

For the test phase, patches were extracted from each painting using the same sliding-window procedure (see Figure 4, Steps 4 and 5). The trained CNN then classified each patch individually, yielding class probabilities (“Veronese” or “Non-Veronese”) (see Figure 4, Step 6). The final painting-level prediction was obtained by averaging the probabilities across all patches. Additionally, these patch probabilities were used to generate a Veronese probability heatmap, and overlaying this map onto the original painting yields a visual representation of the spatial probability distribution across the artwork (see Figure 4, Step 7). This approach ensures that the final decision is grounded in the painting’s global stylistic consistency and mitigates the influence of localized or anomalous regions.

#### 3.2.2 CNN architecture and configuration

To classify the image patches as either “Veronese” or “Non-Veronese,” a custom CNN architecture was designed, tailored to accommodate the dataset constraints and the nature of the input data. The network input consists of 64 × 64-pixel patches with five channels. This multichannel input allows the model to leverage color, structural, and edge features simultaneously, enhancing its ability to capture stylistic nuances.

The CNN architecture comprises two convolutional blocks followed by a fully connected layer and a softmax output layer (see Table 2 for details). Each convolutional block includes a convolutional layer with a 3 × 3 kernel, batch normalization (Ioffe and Szegedy, 2015), and a ReLU activation (Nair and Hinton, 2010). Max-pooling layers with a stride of 2 are applied to progressively reduce spatial dimensions while retaining the most salient features (Krizhevsky et al., 2017). A dropout layer with a rate of 0.5 is applied after the second convolutional block to mitigate overfitting, particularly given the high redundancy introduced by overlapping patches (Srivastava et al., 2014). The final fully connected layer maps the extracted features to the two output classes, and the softmax layer yields class probabilities.

**Table 2:** CNN architecture and configuration: layers, their types, parameters, and output sizes.

Layer	Type	Parameters	Output size
1	Image Input	64 × 64 × 5, Normalization: None	64 × 64 × 5
2	Convolution 2D	3 × 3 kernel, 16 filters, Padding: same	64 × 64 × 16
3	Batch Normalization	–	64 × 64 × 16
4	ReLU	–	64 × 64 × 16
5	Max Pooling 2D	2 × 2, Stride: 2	32 × 32 × 16
6	Convolution 2D	3 × 3 kernel, 32 filters, Padding: same	32 × 32 × 32
7	Batch Normalization	–	32 × 32 × 32
8	ReLU	–	32 × 32 × 32
9	Max Pooling	2 × 2, Stride: 2	16 × 16 × 32
10	Dropout	Rate: 0.5	16 × 16 × 32
11	Fully Connected	2 neurons	2
12	Softmax	–	2

The CNN was trained with the Adam optimizer (Kingma and Ba, 2014), which combines adaptive learning-rates updates with momentum to accelerate convergence and improve training stability (Qian, 1999). Training ran for up to 100 epochs, with a mini-batch size of 128 to balance computational efficiency and gradient estimation accuracy. To improve generalization, the training data were shuffled before each epoch, reducing the risk of learning spurious correlations. To mitigate overfitting caused by the high degree of patch overlap, L2 regularization (Krogh and Hertz, 1991) with a factor of 0.0005 was applied to the network weights. This penalizes large weights, encouraging simpler models that generalize better. CNN’s performance was assessed on a separate validation set at the end of each epoch. Early stopping (patience = 8 epochs) halted training if the validation loss failed to improve for 8 consecutive epochs (Prechelt, 2012). Finally, the checkpoint that achieved the best validation performance was retained as the final model.

#### 3.2.3 CNN validation and testing

To ensure a rigorous evaluation of the CNN, a 6-fold cross-validation strategy was implemented at the painting level (leave-one-painting-out), rather than at the patch level, to avoid bias and data leakage caused by high patch overlap. In each fold, the patches of five paintings were used for training, and the remaining painting for validation. The model achieving the highest validation performance in each fold was retained as the fold’s final model. After completing all folds, the results were aggregated, and performance metrics, including precision, sensitivity (recall), specificity, and F1 score, were computed from the aggregated confusion matrix. In addition to these standard metrics, the area under the ROC curve (AUC), the geometric mean (G-mean), which balances sensitivity and specificity and is particularly relevant for imbalanced datasets (Kuncheva et al., 2019), and Cohen’s kappa which accounts for agreement beyond chance and provides a more robust assessment of classification reliability (Warrens, 2014) were also computed to assess the model’s discriminative ability and reliability.

To evaluate the model’s performance beyond cross-validation, a two-part test procedure was performed. First, the final CNN was trained with all patches from the six paintings in the training dataset, reserving 5% of these patches as a hold-out validation set to apply early stopping with the same criteria applied during cross-validation (patience = 8 epochs). This model was then tested on the test dataset (see Figure 3) composed exclusively of non-Veronese paintings, allowing assessment of its ability to correctly reject non-authentic works. The second part of the procedure evaluated the model’s capacity to recognize authentic Veronese paintings: each of the four Veronese paintings in the training dataset (see Figure 2) was used as a test case in turn, while the CNN was retrained after excluding the corresponding painting from the training set. The CNN produced class probabilities for each patch, and the final painting-level classification was obtained by averaging the probabilities across all the patches. These patch probabilities were used to generate Veronese probability heatmaps for visual analysis.

Finally, the painting under examination was evaluated with the final CNN model trained on all six paintings in the training dataset. The model classified its patches individually. The final painting-level score was obtained by averaging the Veronese probabilities across all patches, reflecting the likelihood that the work is an authentic Veronese. As with the test paintings, these patch probabilities were used to generate a Veronese probability heatmap for detailed visual inspection.

The complete MATLAB R2025b source code, which implements the end-to-end proposed methodology for CNN training and testing, along with the full training and test datasets, is publicly available at https://www.ugr.es/~demiras/PaintingAuthentication/.

## 4 Results

This section presents the experimental results, first detailing the CNN’s cross-validation performance (Section 4.1), then the authentication results for the test paintings and the painting under investigation (Section 4.2), and finally the comparison with the baseline model MobileNetV2 (Section 4.3).

### 4.1 CNN validation results

The experimental setup described above is summarized as follows: the training dataset consisted of six paintings (four by Veronese and two from his workshop, see Figure 2), each cropped and resized to 640 × 640 pixels. From each painting, patches were extracted using a sliding window procedure (64 × 64 pixels, 65% overlap) applied to a 5-channel input stack created by combining the three representations: RGB (3 channels), grayscale (1 channel), and edge map (1 channel). This process generated 4,374 patches (729 per painting), with each patch having dimensions of 64 × 64 × 5. Finally, the CNN was evaluated with 6-fold, painting-level cross-validation (leave-one-painting-out) to prevent data leakage and ensure a robust assessment of generalization.

**Figure 5:**
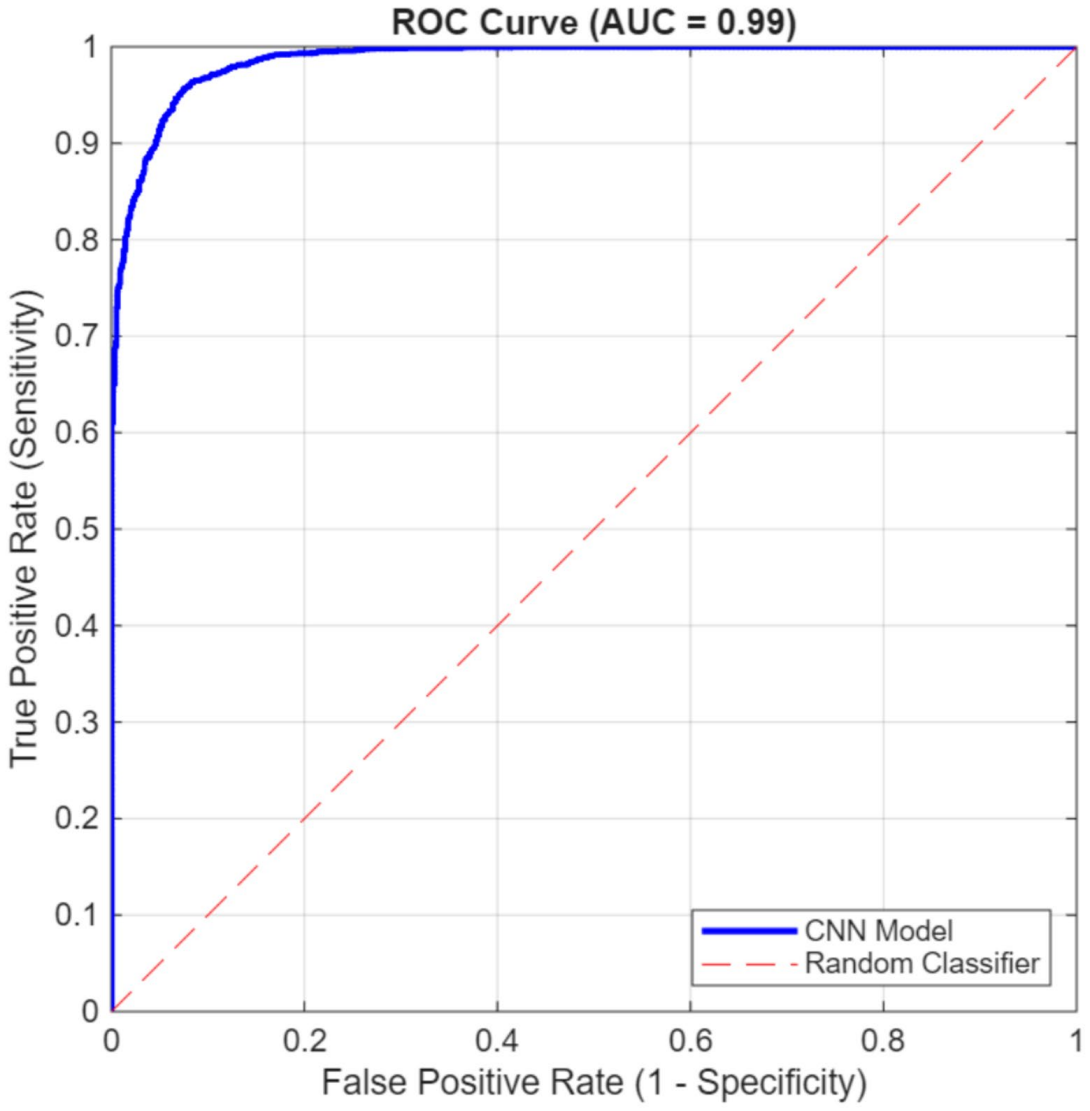
ROC curve and AUC for the CNN from the 6-fold, painting-level cross-validation.

Table 3 reports the confusion matrix aggregated across all six painting-level folds. The key performance metrics derived from this matrix are summarized in Table 4. Finally, Figure 5 depicts the corresponding ROC curve and the resulting AUC.

**Table 3:** Confusion matrix of the CNN model’s performance across the validation folds.

Actual / Predicted	Predicted Veronese	Predicted non-Veronese	Actual total
Actual Veronese	2,833 (TP)	157 (FN)	2,990
Actual non-Veronese	83 (FP)	1,301 (TN)	1,384
Predicted total	2,916	1,458	4,374

Values represent the total count of patches aggregated across all six cross-validation folds. TP, True positive; FN, False negative; FP, False positive; TN, True negative.

**Table 4:** CNN performance metrics aggregated across all six-painting level validation folds.

Accuracy	Precision	F1-score	Sensitivity	Specificity	G-mean	Kappa
94.51%	94.75%	95.94%	97.15%	89.23%	93.11%	0.87

As detailed in Tables 3, 4, the CNN demonstrates high accuracy and strong agreement beyond chance. The slightly lower specificity indicates that classifying non-Veronese patches is more challenging. The model’s overall discriminative power is exceptional, as evidenced by an AUC of 0.99 (see Figure 5).

### 4.2 CNN testing and authentication results

The CNN was tested on the test dataset (see Figure 3) composed exclusively of non-Veronese paintings. Additionally, to evaluate its capacity to recognize authentic Veronese paintings, a leave-one-out approach was used: each of the four Veronese paintings in the training dataset (see Figure 2) was used as a test case in turn, while the CNN was retrained after excluding that painting from the training set. Because the goal is to authenticate a painting, the test phase prioretized analysis of the probability distribution of the Veronese class across each painting rather than computing standard performance metrics. For each test painting, the CNN produced a class probability for every patch. These scores were then aggregated by averaging to obtain a painting-level authenticity score for the entire painting. This approach also enabled a spatial visualization of the model’s predictions in the form of probability heatmaps.

**Figure 6:**
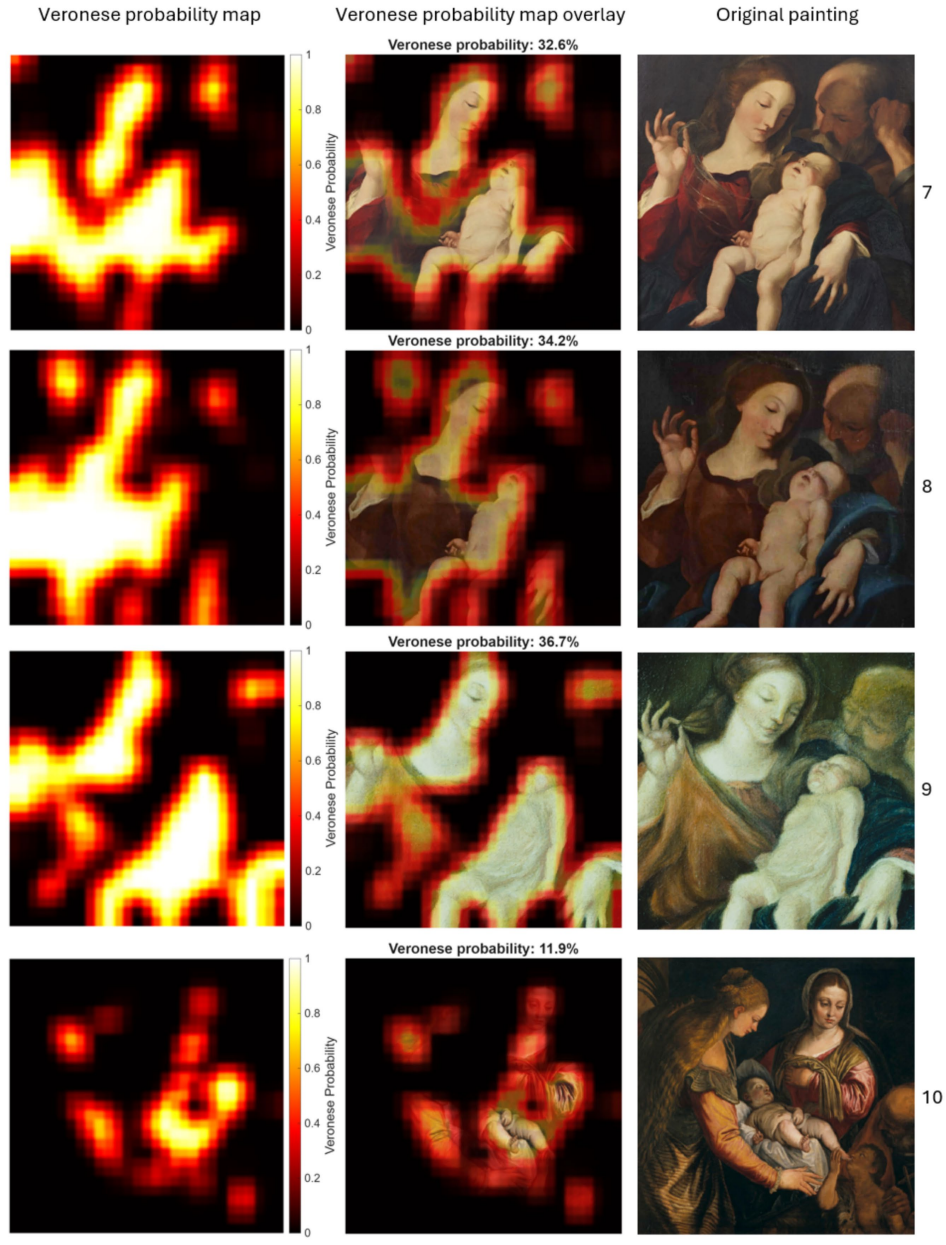
Localized Veronese probabilities for patches in the test dataset of non-Veronese paintings. The columns display: Veronese probability maps (left), probability map overlays with global average probabilities (middle), and the original paintings (right).

Figure 6 shows the results for the test dataset of non-Veronese paintings, displaying both the patch-level Veronese probability heatmap and its overlay on the original image. The heatmap was overlaid on the original painting using alpha blending, with transparency set directly proportional to the Veronese probability. As expected, these works exhibit low Veronese probabilities, with painting-level average Veronese probabilities ranging from 11.9 to 36.7%. This low score is particularly noteworthy for Painting 10, which is a direct copy of authentic Painting 4 (which was included in the training set). Despite sharing an identical composition, the model correctly assigned the copy the lowest Veronese probability (11.9%), demonstrating its ability to distinguish the master’s original technique from a reproduction. Analysis of patch-level variance showed that the 95% confidence-interval upper bounds for all non-Veronese copies consistently remained below 40% (specifically, the highest upper bound was 39.93% for Painting 9), indicating a statistically significant separation from the authenticated painting (see results below). The Veronese probability heatmaps display sparse, fragmented regions of high probability concentrated around localized details, reflecting limited stylistic alignment.

**Figure 7:**
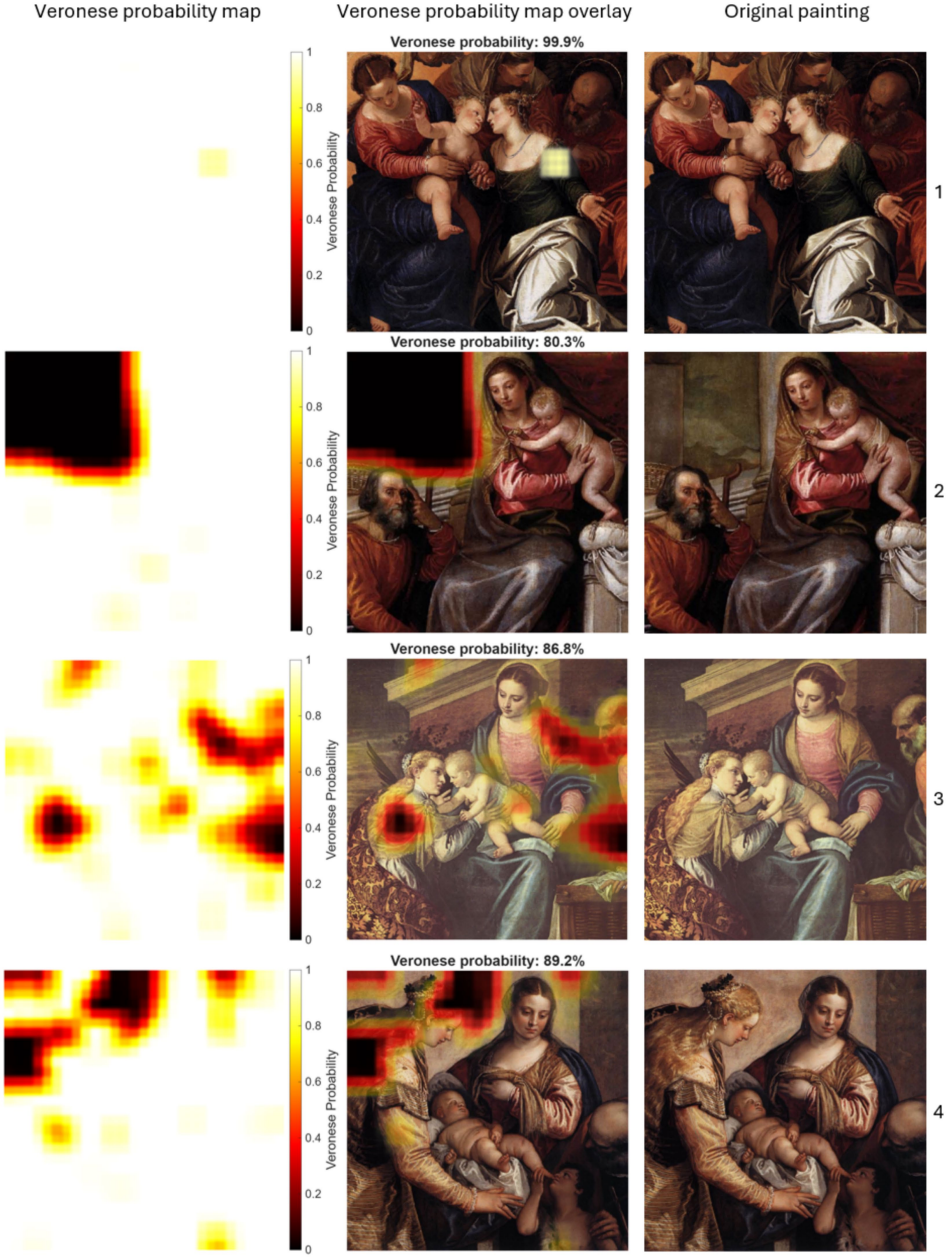
Localized Veronese probabilities for patches in authentic Veronese paintings. The columns show: Veronese probability maps (left), probability map overlays with global average probabilities (middle), and the original paintings (right).

Figure 7 reports the results for the four authentic Veronese paintings. All authentic paintings achieved high painting-level average probabilities (ranging from 80.3 to 99.9%), demonstrating the model’s ability to generalize to unseen authentic paintings. Even accounting for patch-level variance, 95% confidence-interval lower bounds for all authentic paintings exceeded 77% (the lowest being 77.45% for Painting 2), maintaining a clear margin above the upper bounds for non-Veronese paintings. The Veronese probability heatmaps corroborate this finding, showing extensive high Veronese probability regions across all key compositional areas.

**Figure 8:**
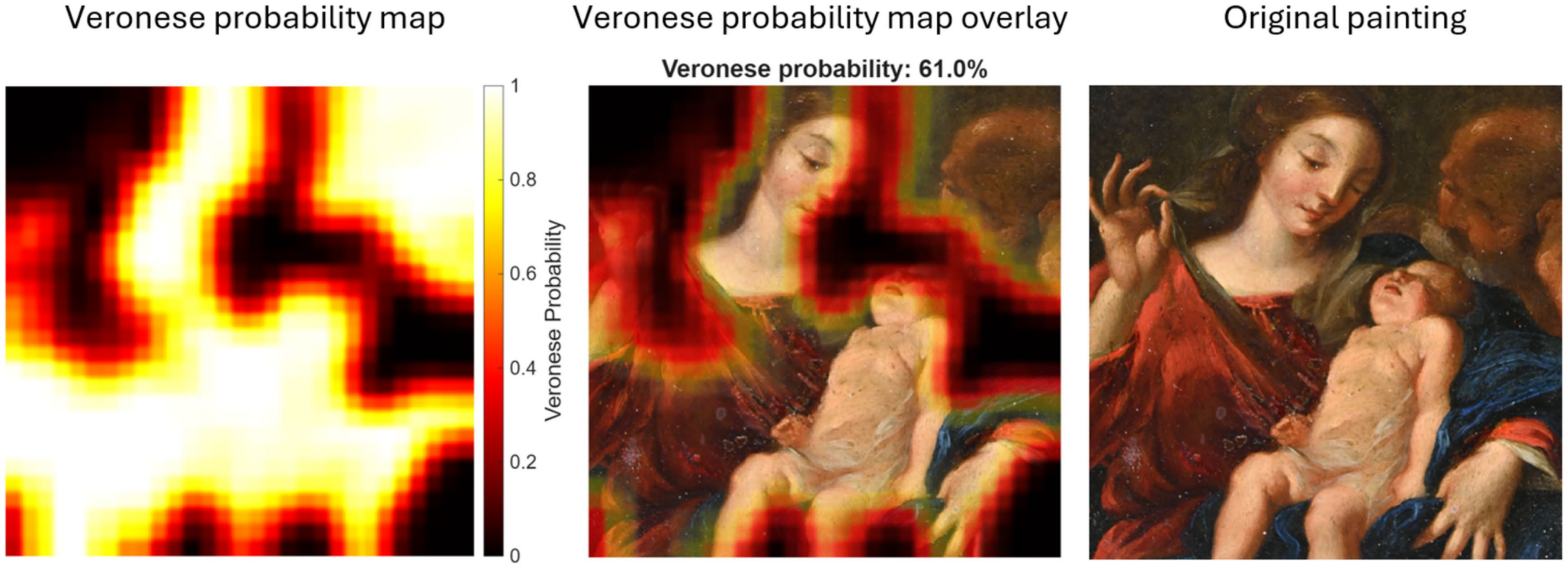
Localized Veronese probabilities for patches in the painting under authentication: Veronese probability map (left), overlay of probability map with global average probability (middle), and original painting (right).

Finally, Figure 8 reports the results for the painting under authentication. Regions of high Veronese likelihood concentrate primarily in areas containing anatomical details and drapery folds. The painting-level average Veronese probability was 61.0%. The standard deviation across the patches was 0.41, resulting in a 95% confidence interval (CI) for the mean of [58.00, 63.96%]. This interval falls within a distinct intermediate range, separated from the upper bound of the non-Veronese copies (< 40%) and the lower bound of the authentic works (> 77%). This intermediate value suggests a moderate stylistic alignment with authentic Veronese’s paintings, indicating that although the painting shares relevant stylistic features with authentic Veronese works, the evidence remains insufficient for full attribution.

### 4.3 Comparison with baseline model (MobileNetV2)

To benchmark the proposed shallow architecture against a state-of-the-art model, a comparative evaluation was conducted using MobileNetV2 (Sandler et al., 2018) via transfer learning. Since the pretrained architecture of MobileNetV2 expects 224 × 224-pixel RGB (3 channels) inputs, the experimental setup was adapted accordingly: patches were upsampled from 64 × 64 to 224 × 224 using bicubic interpolation, and the grayscale and edge channels were discarded. Table 5 summarizes comparative performance.

**Table 5:** Performance comparison of the proposed shallow CNN and MobileNetV2.

Data	Proposed shallow CNN	MobileNetV2 (transfer learning)
Input	64 × 64 × 5	224 × 224 × 3
Parameters	21,800	2,200,000
Cross-validation		
Accuracy	94.51%	97.23%
Precision	94.75%	98.61%
F1-score	95.94%	97.91%
Sensitivity	97.15%	97.22%
Specificity	89.23%	97.26%
G-mean	93.11%	97.24%
Kappa	0.87	0.94
AUC-ROC	0.99	0.99
Test (% Veronese probability)		
Painting 1 (Veronese)	99.9 (99.7–100.0)	72.8 (72.6–73.0)
Painting 2 (Veronese)	80.3 (77.5–83.1)	73.0 (72.9–73.1)
Painting 3 (Veronese)	86.8 (84.9–88.8)	62.8 (61.6–64.0)
Painting 4 (Veronese)	89.2 (87.2–91.2)	69.0 (68.4–69.7)
Painting 7 (non-Veronese)	32.6 (29.6–35.6)	56.1 (54.7–57.4)
Painting 8 (non-Veronese)	34.2 (31.2–37.2)	72.7 (72.5–72.9)
Painting 9 (non-Veronese)	36.7 (33.6–39.9)	34.3 (33.2–35.3)
Painting 10 (non-Veronese)	11.9 (10.0–13.8)	71.7 (71.3–72.1)
Painting under authentication	61.0 (58.0–64.0)	63.6 (62.4–64.8)

Veronese probabilities are reported as mean (95% confidence interval).

In 6-fold cross-validation, MobileNetV2 demonstrated strong performance, achieving an accuracy of 97.23%, precision of 98.61%, F1 score of 97.91%, sensitivity of 97.22%, specificity of 97.26%, G-mean of 97.24%, and a Kappa coefficient of 0.94. The AUC-ROC was 0.99. These results slightly surpassed those of the proposed shallow CNN during cross-validation.

However, despite strong cross-validation metrics, the model exhibited poor generalization on the test dataset. MobileNetV2 failed to establish a decision boundary between non-Veronese paintings and authentic Veronese works: (1) it assigned high Veronese probabilities to non-Veronese samples, such as 72.7% (Painting 8) and 71.7% (Painting 10), resulting in substantial false positives compared to the proposed model (< 37%); and (2) conversely, probabilities for authentic Veronese paintings were lower than expected with MobileNetV2 (62.8–73.0%), indicating reduced sensitivity relative to the proposed model (> 80%). For the painting under authentication, MobileNetV2 yielded a Veronese probability of 63.6%.

## 5 Discussion and conclusions

This study addressed the challenge of authenticating paintings under conditions of data scarcity by developing a patch-based CNN pipeline tailored to stylistic analysis. The methodology combined sliding window feature extraction with multichannel inputs (RGB, grayscale, and edge maps) to capture complementary visual cues and implemented painting-level cross-validation to prevent data leakage. By expanding the dataset with localized patches and integrating regularization strategies, the approach aimed to enhance generalization while preserving stylistic fidelity. The CNN trained with these patches distinguished authentic Veronese works from non-Veronese paintings at both painting- and patch-levels. Authentic Veronese paintings show high painting-level probabilities (ranging from 80.3 to 99.9%) and Veronese probability heatmaps with broad, contiguous high-probability regions (see Figure 7), whereas non-Veronese paintings exhibit lower painting-level scores (ranging from 11.9 to 36.7%) and fragmented, localized regions of high probability (see Figure 6). The painting under authentication yields an intermediate painting-level score (61.0%) with extensive high-probability zones over stylistically salient passages, interspersed with some lower-probability areas (see Figure 8).

The sliding window design (64 × 64 patches, 65% overlap) expanded the training data from a small number of paintings to thousands of localized samples, encouraging the CNN to learn microtextural and brushwork signatures while controlling overfitting via dropout, L2 regularization (weight decay), batch normalization, the Adam optimizer, and early stopping. The high overlap mitigates boundary artifacts and stabilizes local predictions, an effect analogous to the overlap-tile inference widely used in biomedical image analysis (Ronneberger et al., 2015). The multichannel design (RGB, grayscale, and edge map) leveraged complementary cues: chromatic information, luminance-texture structure, and gradient-defined contours. The Canny edge detector is well-suited here due to its double-threshold mechanism and robustness to noise, which supports capturing subtle, style-relevant edge patterns (Canny, 1986).

The 6-fold painting-level cross-validation was essential to prevent data leakage from overlapping patches, an issue known to inflate performance in tile-based pipelines and subject-repeated imaging datasets (Bussola et al., 2019; Rumala, 2023). Cross-validation confirmed strong overall performance (see Table 4; Figure 5): accuracy 94.51%; precision 94.75%; F1 score 95.94%; sensitivity 97.15%; specificity 89.23%; G-mean 93.11%; Cohen’s kappa 0.87; and AUC-ROC 0.99. This set of metrics provides a balanced view: AUC-ROC summarizes rank discrimination (Fawcett, 2006); G-mean emphasizes balanced sensitivity and specificity under class imbalance (Kuncheva et al., 2019); and Cohen’s kappa quantifies agreement beyond chance (Warrens, 2014). The validation metrics confirmed the CNN model’s high accuracy and exceptional discriminative power. The model was particularly effective at identifying authentic Veronese paintings (sensitivity = 97.15%), with slightly lower performance for non-Veronese paintings (specificity = 89.23%).

The heatmap visualization of Veronese probability provides critical insight into CNN’s decision-making process. In the painting under authentication (see Figure 8), high-probability regions cluster around stylistically salient elements such as anatomical contours and tonal transitions, whereas low-probability regions dominate less textured or peripheral areas. This uneven distribution suggests that the model detects Veronese-like features selectively rather than uniformly across the composition, helping to explain the intermediate global score of 61%. From a methodological perspective, this outcome reflects the patch-based classification strategy: aggregate probabilities capture local stylistic heterogeneity rather than enforce global uniformity. From an interpretive standpoint, the observed pattern may indicate (1) an authentic work with workshop participation; (2) an authentic work with later interventions or restoration that may alter original textures; or (3) a high-quality workshop production under close supervision. Consequently, while the CNN identifies meaningful stylistic affinities, the evidence remains insufficient to support definitive attribution.

The comparison between the painting under authentication and the non-Veronese replicas of the composition (paintings 7, 8, and 9) highlights the CNN’s capacity to assess stylistic consistency at a global level while acknowledging local similarities. Although the non-Veronese paintings exhibit localized high-probability regions (see Figure 6), primarily around anatomical details and drapery folds, their overall scores remain low (ranging from 32 to 37%), indicating limited stylistic coherence. In contrast, the painting under authentication shows broader regions of high Veronese probability and a substantially higher global score (61%), suggesting a more pervasive presence of Veronese-like features. This pattern supports the hypothesis of partial authenticity or strong stylistic influence for the painting under authentication and confirms that the CNN does not rely solely on compositional similarity but captures nuanced textural and structural cues. The residual probabilities in non-Veronese paintings may arise from shared iconographic elements, underscoring the importance of interpreting probability heatmaps in conjunction with global metrics rather than in isolation. A key detail is that in all non-Veronese paintings, the area corresponding to St. Joseph in the upper-right corner consistently yields very low probabilities, whereas in the painting under authentication the corresponding region exhibits high probabilities (see Figure 8). This contrast indicates that the CNN captures subtle stylistic cues in localized passages that are absent or less pronounced in non-Veronese paintings despite compositional similarity.

The probability heatmaps for authentic Veronese paintings (see Figure 7) reveal broad, continuous regions of high probability. This spatially cohesive distribution contrasts sharply with the fragmented patterns observed in non-Veronese works and supports the CNN’s capacity to capture stylistic coherence that extends beyond isolated details. The consistently high painting-level scores for Veronese paintings (ranging from 80.3 to 99.9%) indicate that the model generalizes effectively to unseen authentic works. These findings support the patch-based approach as a robust strategy for identifying nuanced textural and structural cues characteristic of Veronese’s technique.

The comparison between paintings 4 (authentic Veronese) and 10 (copy) is particularly noteworthy. Despite compositional similarity, the authentic work yields a high painting-level probability (89.2%) and extensive high-probability regions (Figure 7). In contrast, the copy yields a low painting-level probability (11.9%) with sparse and fragmented high-probability regions (Figure 6). This supports the CNN’s capacity to differentiate stylistic coherence from superficial compositional similarity.

Notably, one authentic Veronese painting (see painting 2 in Figure 7) exhibits a sharply defined low-probability region confined to a rectangular area in the upper-left corner. Two nonexclusive explanations are plausible: (1) the model may struggle to classify uniform, low-texture passages that provide weak class-specific signals; (2) such areas may indicate workshop participation in secondary passages, an documented practice in 16th-century Venice in which masters concentrated on principal figures while assistants executed peripheral and ornamental elements (Gisolfi, 2017).

To comprehensively benchmark the proposed architecture, a comparative experiment with MobileNetV2 (Sandler et al., 2018) via transfer learning using a 224 × 224 × 3 input was conducted. The results indicated critical limitations in applying standard deep learning models to this particular domain. Although MobileNetV2 achieved high metrics during cross-validation (accuracy: 97.23%), it failed to establish a discriminative boundary in the test dataset, yielding overlapping probability ranges to Veronese works (62.8–73.0%) and non-Veronese paintings (up to 72.7%). Two technical factors account for this generalization failure when compared with the proposed architecture. First, the loss of resolution: the required upsampling from 64 × 64 to 224 × 224 pixels effectively acts as a low-pass filter, smoothing out high-frequency microtextures essential for distinguishing Veronese brushwork. Consequently, the pretrained model likely overfitted to macroscopic features, such as color palettes and semantic content and composition, shared by both originals and workshop productions. Second, input rigidity: by restricting the input to RGB channels, MobileNetV2 excluded the explicit grayscale intensity and edge maps used in the proposed pipeline, thereby losing critical topological information about brushstroke dynamics that proved decisive in the shallow CNN.

Notably, for the Holy Family painting under authentication, MobileNetV2 predicted a Veronese probability of 63.6%, closely matching the 61.0% obtained by the proposed shallow CNN. This convergence suggests that the classification of the artwork as an intermediate or workshop production is a robust signal, persisting across different model architectures. Although MobileNetV2 lacks sufficient specificity to reject clearly non-Veronese works, its agreement with the proposed specialized shallow CNN for the disputed artwork supports the reliability of the assessment: the painting exhibits a hybrid visual structure that is neither accepted as a fully authentic Veronese work nor rejected as a mere Veronese-style replica.

### 5.1 Limitations

While the CNN pipeline developed in this study demonstrates strong performance in distinguishing authentic Veronese paintings from non-Veronese works, several limitations must be acknowledged, particularly for the authentication of a single, stylistically ambiguous painting. (1) The dataset is narrowly focused on a specific iconographic theme, representations of the Holy Family. While this restriction was deliberate to minimize semantic noise and encourage the model to focus on painterly execution rather than composition, it also limits the generalizability of the learned features. The model may have become particularly sensitive to iconography-specific details, such as poses, color schemes, or thematic composition common to religious scenes, rather than capturing a general Veronese style applicable across other genres like portraiture and mythological scenes. Consequently, this constraint indicates that the model is designed for a specific authentication task rather than for general classification across Veronese’s broader body of work. (2) The patch-based approach, while effective for capturing localized stylistic features, inherently limits learning on higher-order stylistic structures such as overall composition, figure-scale relationships, and macro-level color organization. As a result, the authentication lacks the holistic structural analysis typically undertaken by art historians. Conversely, this constraint enables the model to detect execution differences in compositionally identical copies (e.g., Painting 10). (3) The binary classification framework (Veronese versus non-Veronese) reflects limited availability of comparable works and does not account for intermediate cases such as workshop productions or restorations. Future research should extend this framework by incorporating distinct classes such as “Workshop of Veronese,” “In the style of,” and “Later additions,” contingent on sufficient training data becoming available. A multiclass approach would better reflect the complex reality of Renaissance artistic production, enabling the model to distinguish between the master’s autograph execution and collaborative production typical of his workshop. (4) Although the CNN offers interpretable probability heatmaps, these visualizations are primarily qualitative and can be affected by patch overlap and boundary artifacts. (5) Finally, while the model identifies stylistic affinities with high sensitivity, its output should be interpreted as probabilistic evidence rather than as definitive attribution.

### 5.2 Conclusion

This study demonstrates that the challenge of extreme data scarcity in art authentication can be effectively addressed with tailored computational strategies. By reducing the network complexity to align with the limited historical record, the proposed, shallow, custom architecture achieved robust generalization (accuracy: 94.51%, AUC-ROC: 0.99) and higher specificity than standard solutions. The comparative experiment showed that large-scale pretrained models like MobileNetV2, while performing well during cross-validation, fail to reliably distinguish non-Veronese paintings from originals. These results support the premise that for specific heritage tasks, a tailored, texture-focused model is not only more resource-efficient but also diagnostically more reliable than general-purpose large-scale models.

For the Holy Family painting under examination, the proposed model provides strong quantitative evidence against a binary authentic-versus-inauthentic classification. The painting-level probability of 61.0%, supported by a tight confidence interval (58.0–64.0%), places the artwork in a statistically distinct category, separated from the non-Veronese paintings (< 40%) and authentic Veronese works (> 77%). These results support the hypothesis of either high-quality workshop production or partial authorship, providing a level of nuance that subjective visual inspection alone cannot quantify. Therefore, conclusive authentication will require further integration of technical, historical, and provenance analyses beyond computational inference alone.

## Data Availability

The datasets and source code in this study are publicly available at https://www.ugr.es/~demiras/PaintingAuthentication/.

## Footnotes

1
https://www.wikiart.org/
