# Correction: Painting authentication using CNNs and sliding window feature extraction

**DOI:** 10.3389/frai.2026.1818182

**Published:** 2026-03-13

**Authors:** Juan Ruiz de Miras, José Luis Vílchez, María José Gacto, Domingo Martín

**Affiliations:** 1 Software Engineering Department, University of Granada, Granada, Spain; 2 Department of Analytical Chemistry, University of Granada, Granada, Spain; 3 Biosanitary Research Institute (ibs.GRANADA), Granada, Spain; 4 Andalusian Research Institute in Data Science and Computational Intelligence (DaSCI), University of Granada, Granada, Spain

**Keywords:** authentication, convolutional neural networks, painting classification, Paolo Veronese, sliding-window patch extraction

An incorrect **Funding** statement was provided. The correct funder is Spanish Ministry of Science, Innovation and University MICIU/AEI/10.13039/501100011033 and FEDER EU.

The correct funding statement reads:

The author(s) declared that financial support was received for this work and/or its publication. This research was partially funded by the Spanish Ministry of Science, Innovation and University MICIU/AEI/10.13039/501100011033 and FEDER EU (grant no. PID2024-161348OB-I00), and the Carlos III Health Institute, co-funded by the European Union and ERDF A way of making Europe (grant no. PI23/00129).

The original version of this article has been updated.

